# Liver Toxicity of Cadmium Telluride Quantum Dots (CdTe QDs) Due to Oxidative Stress *in Vitro* and *in Vivo*

**DOI:** 10.3390/ijms161023279

**Published:** 2015-09-25

**Authors:** Ting Zhang, Yuanyuan Hu, Meng Tang, Lu Kong, Jiali Ying, Tianshu Wu, Yuying Xue, Yuepu Pu

**Affiliations:** 1 Key Laboratory of Environmental Medicine Engineering, Ministry of Education, School of Public Health, Southeast University, Nanjing 210009, China; E-Mails: zhangting1207@gmail.com (T.Z.); huyuanyuan1122@gmail.com (Y.H.); konglu@seu.edu.cn (L.K.); yingjiali1020@163.com (J.Y.); ninatswu@126.com (T.W.); yyxue@seu.edu.cn (Y.X.); 2 Jiangsu Key Laboratory for Biomaterials and Devices, Southeast University, Nanjing 210009, China

**Keywords:** quantum dots, oxidative stress, Nrf2, toxicity mechanisms

## Abstract

With the applications of quantum dots (QDs) expanding, many studies have described the potential adverse effects of QDs, yet little attention has been paid to potential toxicity of QDs in the liver. The aim of this study was to investigate the effects of cadmium telluride (CdTe) QDs in mice and murine hepatoma cells alpha mouse liver 12 (AML 12). CdTe QDs administration significantly increased the level of lipid peroxides marker malondialdehyde (MDA) in the livers of treated mice. Furthermore, CdTe QDs caused cytotoxicity in AML 12 cells in a dose- and time-dependent manner, which was likely mediated through the generation of reactive oxygen species (ROS) and the induction of apoptosis. An increase in ROS generation with a concomitant increase in the gene expression of the tumor suppressor gene *p53*, the pro-apoptotic gene *Bcl-2* and a decrease in the anti-apoptosis gene *Bax*, suggested that a mitochondria mediated pathway was involved in CdTe QDs’ induced apoptosis. Finally, we showed that NF-E2-related factor 2 (Nrf2) deficiency blocked induced oxidative stress to protect cells from injury induced by CdTe QDs. These findings provide insights into the regulatory mechanisms involved in the activation of Nrf2 signaling that confers protection against CdTe QDs-induced apoptosis in hepatocytes.

## 1. Introduction

With the development of nanotechnology, numerous nanomaterials have been created for a variety of applications. Quantum dots (QDs) are one of the most promising developments among these nanomaterials, because their unique autofluorescence properties have great potential in biomedicine for diagnosis, drug delivery, and imaging [1]. Although QDs have great potential in terms of clinical applications, they have been shown to cause adverse effects *in vitro* and *in vivo* and have potential risks for human health. QDs can be distributed to all body systems and can be aggregated in some tissues and organs [2,3,4]. QDs deposited in cells can cause cell viability and morphology change, including reducing cell viability, inducing chromatin condensation, and inducing apoptosis [5,6,7,8].

The assessment of the health risk of QDs to the human body is based upon the level of exposure, the toxicity of the material in question, the route of exposure and the persistence of the particular material in the organism. To evaluate the toxic effects of QDs, it is important to understand the biodistribution of QDs. A number of studies have investigated the biodistribution of QDs and their whole body clearance after intravenous administration, and found that the liver is to be a major accumulation site for circulatory QDs [4,9]. The liver is the key organ for detoxification of xenobiotics by metabolism and biliary excretion. Small molecules, after intravenous injection, are normally distributed in the liver sinusoids and then taken up by hepatocytes by passive diffusion or through various transporters, with subsequent metabolism by various liver enzymes [10]. The liver is characterized by its distinct populations of cells, each with their own unique morphology and a wide arrays of functions that are crucial in overall liver activity [11,12]. Therefore, understanding the toxicity mechanism of QDs in the liver and its hepatocytes is critical. A recent study found that cadmium selenide (CdSe) QDs were readily distributed into various organs upon their *in vivo* administration and the liver appeared to be the predominant site for the QD accumulation. The administered CdSe QDs also induced dramatic hepatic toxicity *in vivo* and *in vitro* [13]*.* Additionally, *in vitro* studies with cadmium telluride (CdTe) QDs also demonstrated their ability to induce cytotoxicity, oxidative stress and apoptosis in HepG2 cells [14,15].

There is much evidence suggesting the involvement of oxidative stress in QD-induced toxicity [16]. Reactive oxygen species (ROS) and other free radicals are critical intermediates in the normal physiology and pathophysiology of the liver [17]. However, excess production of ROS and the impairment of antioxidant defense is the underlying mechanism of oxidative stress in cells. Oxidative stress can activate a variety of cellular responses, such as DNA oxidative damage, abnormal protein expression, and mitochondrial dysfunction [18], resulting in cell apoptosis [19] and damage to the body [20]. Therefore, how oxidative stress plays a role in QD-induced liver and hepatocyte toxicity *in vivo* and *in vitro* was investigated in the present study.

Because the Nrf2 protein is a master regulator for the expression of multiple antioxidant genes, it is also considered a master regulator of redox homeostasis and plays a central role in antioxidant and anti-inflammatory defense [21]. Activation of the Nrf2 pathway induces a cellular protective system that promotes DNA damage recognition and repair, as well as cell survival, under detrimental environmental conditions. The roles of Nrf2 and the Nrf2-responsive genes involved in cell survival in the nanoparticles-mediated cytotoxicity in intestinal cells are not known, and no sensitive biomarkers for QD-toxicity studies are currently available for hepatocytes.

Previously, it was unclear whether oxidative stress and apoptosis induced by CdTe QDs were related to the Nrf2/ antioxidant response element (ARE) pathway. Therefore, in the present study, we aimed to investigate the adverse effects of CdTe QDs on the liver using mice and AML 12 cells as an *in vivo* and *in vitro* model system, respectively. In addition, we examined the biological effects of exposure to CdTe QDs in AML 12 cells to understand the CdTe QD induced cellular protective mechanism observed in AML 12 cells and clarify if Nrf2 or Nrf2-responsive genes could function as a possible sensitive marker for CdTe QDs-mediated toxicity in the liver.

## 2. Results

### 2.1. Synthesis and Characterization of CdTe QDs

**Figure 1:**
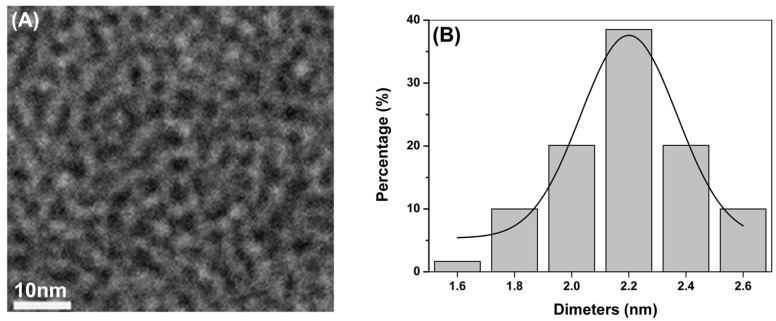

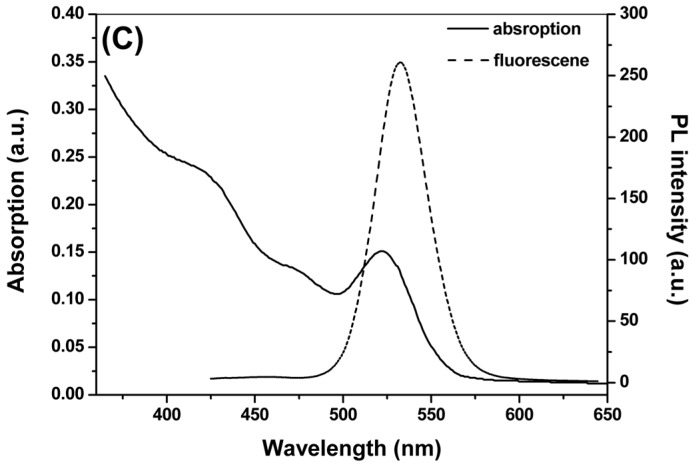
Characterization of CdTe QDs. (**A**) TEM of CdTe QDs; (**B**) Size distribution by dynamic light scattering (DLS) CdTe QDs; (**C**) Absorption and photoluminescence (PL spectrum of CdTe QDs).

The microstructural characterization of the CdTe QDs investigated in this study is summarized in Figure 1 and Table 1. The TEM assessment shows the shape and morphology of the CdTe QDs used in this study (Figure 1A). The analysis based on the nanoparticle size analyzer suggested that the diameter of CdTe QDs was approximately 2.2 nm (Figure 1B). The absorption (solid line) and photoluminescence (PL) (dashed line) spectra of the CdTe QDs was shown in Figure 1C. The excitation absorption peaks of the QDs are quite obvious, and the maximum absorption peaks appeared at 522 nm. The evaluation of the fluorescence spectrum indicated that the maximum emission wavelength was 532 ± 10 nm, and the maximum half width was ≤32 nm. The zeta potentials indicated a negative absolute charge in the CdTe QDs (Table 1).

**Table 1:** Physicochemical characteristics of cadmium telluride quantum dots (CdTe QDs).

Parameters	Values (Mean ± SD)
Hydrodynamic size in distilled water (nm)	9.82 ± 1.14
Hydrodynamic size in culture medium (nm)	26.79 ± 1.59
Zeta potential in distilled water (mV)	−26.46 ± 4.75
Zeta potential in culture medium (mV)	−13.86 ± 3.44

### 2.2. Body Weight, Liver Weight, Histopathological Change and Serum Biochemistry Analysis by 28 Day-Repeated Intravenous Administration of CdTe QDs

Body weight measurements showed no differences between the different dose groups compared and the control group, whereas an increase in the liver weight/body weight coefficient was observed in the 16.5 mg/kg.bw group (Table 2). Substantial CdTe QDs accumulation in the liver might result in injuries to the organ. Therefore, we evaluated tissue changes after chronic exposure at the microscopic level via histological examination. No noticeable alteration was detected in the liver based on histological examination after a 28 day-repeated exposure (data not shown), compared to the vehicle control.

**Table 2:** Animal Weights and Liver/Body Weight (bw) Ratio (final) of mice after 28 days intravenous treatment with CdTe QDs (mg/kg.bw).

Group	Number	Initial Body Weight (g)	Final Body Weight (g)	Liver Weight (g)	Liver/bw Ratio
control	6	19.80 ± 1.48	44.60 ± 2.41	2.60 ± 0.38	5.81 ± 0.58
4.12	6	20.40 ± 1.14	43.40 ± 1.82	2.46 ± 0.14	5.68 ± 0.39
8.25	6	20.40 ± 1.14	42.80 ± 5.58	2.51 ± 0.54	5.83 ± 0.65
16.5	6	20.20 ± 1.48	43.40 ± 2.19	2.17 ± 0.20 *	4.99 ± 0.24 *

*, Values significantly different from control results (*p* < 0.05).

The serum biochemical parameters of the mice were assayed to further evaluate the toxicity of CdTe QDs. Table 3 shows that total protein (TP), total bilirubin (TBIL), direct bilirubin (DBIL), alanine transaminase (ALT), aspartate aminotransferase (AST), γ-glutamyl transpeptidase (γ-GT) and alkaline phosphatase (ALP) levels of all groups were similar after exposure to the CdTe QDs. However, albumin (ALB) levels of the 4.12 mg/kg.bw group and 16.5 mg/kg.bw group were slightly higher than the control group. The lactic dehydrogenase (LDH) levels of all of the exposed group are nearly half of those of the control group.

**Table 3:** Clinical chemistry of mice after 28 days intravenous treatment with CdTe QDs (mg/kg.bw).

Parameters	Concentration of CdTe QDs (mg/kg.bw)			
	4.12	8.25	16.5	NS
TP (g/L)	50.60 ± 3.36	53.60 ± 3.97	53.00 ± 1.73	51.25 ± 2.39
ALB (g/L)	13.60 ± 1.14*	13.40 ± 0.89	13.60 ± 0.89*	12.00 ± 0.71
TBIL (µmol/L)	4.52 ± 0.85	4.78 ± 1.01	3.62 ± 0.41	4.55 ± 0.59
DBIL (µmol/L)	3.52 ± 0.53	4.14 ± 0.94	3.14 ± 0.49	4.06 ± 0.46
ALT (IU/L)	27.00 ± 10.77	58.00 ± 24.82	73.60 ± 59.21	46.00 ± 9.98
AST (IU/L)	97.80 ± 8.26	121.40 ± 50.19	130.80 ± 15.12	124.60 ± 21.34
ALP (IU/L)	100.60 ± 24.69	115.60 ± 45.63	106.80 ± 18.91	89.50 ± 26.37
γ-GT (IU/L)	1.40 ± 0.89	1.80 ± 1.31	1.80 ± 0.84	1.80 ± 0.45
LDH (IU/L)	420.00 ± 88.36 *	540.40 ± 130.34 *	539.00 ± 80.77 *	954.00 ± 247.01

TP, total protein; ALB, albumin; TBIL, total bilirubin; DBIL, direct bilirubin; ALT, alanine transaminase; AST, aspartate aminotransferase; ALP, alkaline phosphatase; γ-GT, γ-glutamyl transpeptidase; LDH, lactic dehydrogenase; NS, normal saline; *,Values significantly different from control results (*p* < 0.05).

### 2.3. Induction of Oxidative Stress in the Liver

**Figure 2:**
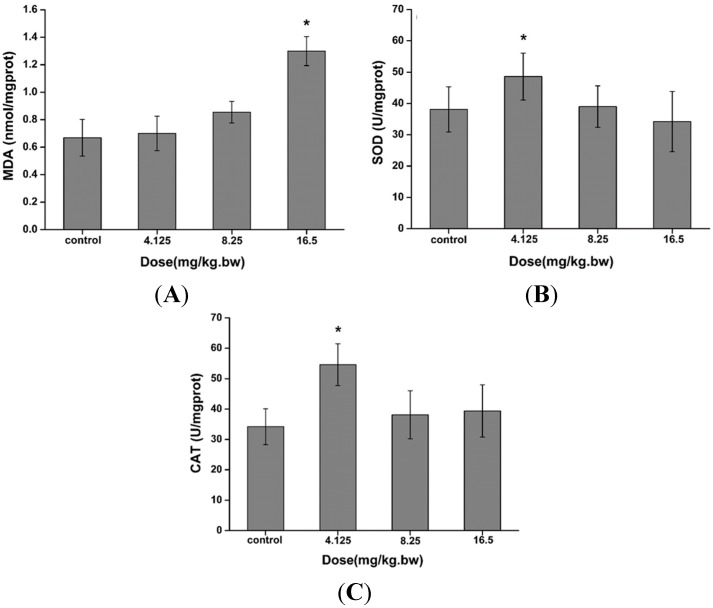
The (**A**) malondialdehyde (MDA) levels; (**B**) superoxide dismutase (SOD) and (**C**) catalase (CAT) activities in the liver after an intratracheal administration with 4.125–16.5 mg/kg.bw CdTe QDs. The asterisks (*) indicate statistically significant differences compared to the control with *p* < 0.05.

Several oxidative stress markers were selected to measure the effects of CdTe QDs on the oxidative status of the liver after intravenous administration of CdTe QDs. The superoxide dismutase (SOD) and catalase (CAT) activities were increased in the 4.12 mg/kg.bw group. The MDA level increased in the 16.5 mg/kg.bw group, reaching a maximum level higher than the control after 28 days of exposure, as indicated in Figure 2. The doubling of MDA concentration may result as a consequence of the cascade mechanism of lipid peroxidation. The obvious change of MDA concentration after 28 days could also be explained by the significant increase of SOD and CAT activities, which are involved in ROS elimination.

### 2.4. Cytotoxicity and ATP Synthesis Induced by CdTe QDs

**Figure 3:**
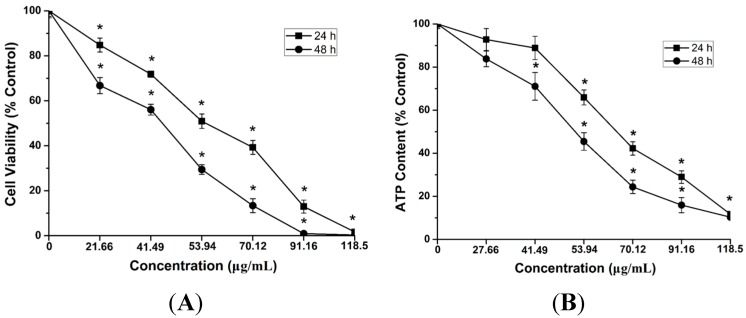
Single-parameter toxicological screening of CdTe QDs in AML12 cells. (**A**) (3-4,5-Dimethyl-2-thiazolyl)-2,5-diphenyl-2*H*-tetrazolium bromide (MTT) assay; and (**B**) ATP assay. AML12 cells were treated with a wide dose range (21.66 to 118.5 μg/mL) of CdTe QDs for 24 and 48 h, respectively. Data are presented as mean ± standard deviations (SD) of the three representative experiments. The asterisks (*) and indicate statistically significant differences compared to control with *p* < 0.05.

To further study the cytotoxicity caused by CdTe QDs at the cellular level, we evaluated cell viability upon CdTe QD exposure by employing *in vitro* cultured cells, murine hepatoma cells AML 12, which might be able to represent hepatocytes from the liver. To examine the potential cytotoxicity of CdTe QDs at different concentrations, AML 12 cells were incubated with different concentrations ranging from 27.66 to 118.5 µg/mL of as-synthesized CdTe QDs. The AML 12 cell viability was determined at 24 and 48 h after treatment. As the exposure dose increased, CdTe QDs significantly inhibited cell growth in AML 12, and the cell viability, revealed by Figure 3A, was decreased by treatment with CdTe QDs in both a time- and concentration-dependent manner. The calculated IC50 value of CdTe QDs was found to be 60.12 μg/mL after the 24 h treatment for AML 12 cells using the method of Bliss [22].

Considering that viability assays are vital steps in toxicology to explain the cellular response to a toxicant and that they provided information on cell death, survival, and metabolic activities, we have exploited the high sensitivity of a luminescence-based assay and a fluorescence-based assay to study the cytoxicity of CdTe QDs in AML 12 cells. The ATP concentration is described as the product of the viable cell number and the average amount of ATP produced by each viable cell. The results showed that the ATP concentration in AML 12 heavily decreased with the increasing treatment concentration of the CdTe QDs (Figure 3B). These results demonstrated that the CdTe QDs can efficiently increase intracellular ATP contents in all the detected cells.

These results indicated that the bioenergy crisis (ATP depletion) should have a great influence on the massive death of AML 12 cells [23].

### 2.5. Reactive Oxygen Species (ROS) Generation by CdTe QDs

**Figure 4:**
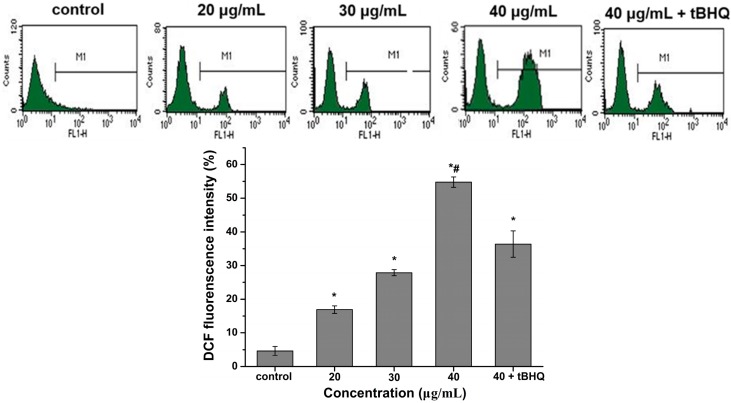
ROS generation in AML12 cells after 24 h of treatment with 20, 30, 40 μg/mL CdTe QDs and (40 μg/mL CdTe QDs + tBHQ). ROS formation was quantified by flow cytometry, and M1 represents the percentage of ROS production. Data are expressed as a fold change in the percentage increase in mean fluorescence intensity (MFI) of dichlorofluorescein (DCF) channel relative to that of untreated cells. The asterisks (*) and indicate statistically significant differences compared to control with *p* < 0.05, respectively. The number (#) signs indicate statistically significant differences compared to the tBHQ pre-treated group (*p* < 0.05).

Oxidative stress is a critical event of cell damage. Many reports have shown induction of ROS mediated cell death in various cells by different nanoparticles. For this study, AML 12 cells were loaded with the ROS measuring probe 20,70-dichlorodihydrofluorescein diacetate (H2DCFDA). As shown in Figure 4, CdTe QDs induced ROS generation in a concentration dependent manner, as indicated by the increase in fluorescence intensity. The highest amount of ROS was generated after 24 h of exposure to 40 μg/mL CdTe QDs, which is a 13-fold increase over untreated control AML 12 cells. At the 40 μg/mL of CdTe QDs exposure, the *tert*-butyl Hydroquinone (tBHQ) intervention group showed a significantly lower incidence of intracellular ROS compared to the nonintervention group (*p* < 0.05). These results show that CdTe QDs can cause elevated levels of ROS in the AML 12 cells in a concentration dependent manner and that antioxidants, such as tBHQ, can reduce intracellular ROS production to a certain extent.

### 2.6. CdTe QDs Nanoparticles Induced Apoptosis

**Figure 5:**
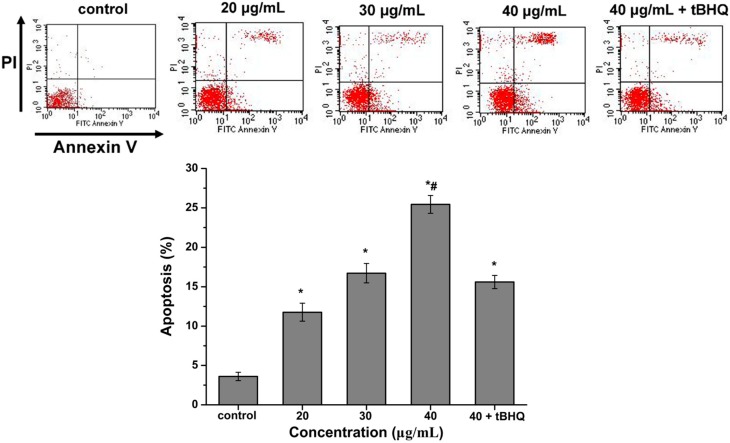
Flow cytometric evaluation of the effect of CdTe QDs on AML12 cells apoptosis and necrosis after 24 h of treatment with 20, 30, 40 μg/mL CdTe QDs and (40 μg/mL CdTe QDs + tBHQ). Cells were stained using apoptotic detection kit (PI/annexin V-FITC). Quadrant P1 shows the percentage of normal viable cells, P2 the percentage of necrotic cells (PI positive cells), P4 the percentage of early apoptotic cells (annexin V-FITC positive cells) and P3 the percentage of cells in its late apoptotic phase (Double positive cells). The asterisks (*) indicate statistically significant differences compared to control with *p* < 0.05, respectively. The number (#) signs indicate statistically significant differences compared to the tBHQ pre-treated group (*p* < 0.05).

Apoptosis is regarded as a general adaptive response when cells are exposed to CdTe QDs *in vitro*. Here, the apoptosis of the cells in organ sections was assayed by using Annexin V-FITC/propidium iodide double-staining and analyzed by flow cytometry. As shown in Figure 5, the percentage of apoptotic cells was 3.61%, 11.77%, 16.72% and 25.44% following treatment with 0, 20, 30 and 40 µg/mL of CdTe QDs, respectively. Flow cytometry showed that as the exposure dose increased, the apoptosis observed in the cells increased significantly over the control group (*p* < 0.05). The tBHQ intervention group, when exposed to the same dose, had a significantly lower incidence of apoptosis (*p* < 0.05).

### 2.7. Effect of CdTe QDs on the Expression of p53, Bax and Bcl-2 mRNA

**Figure 6:**
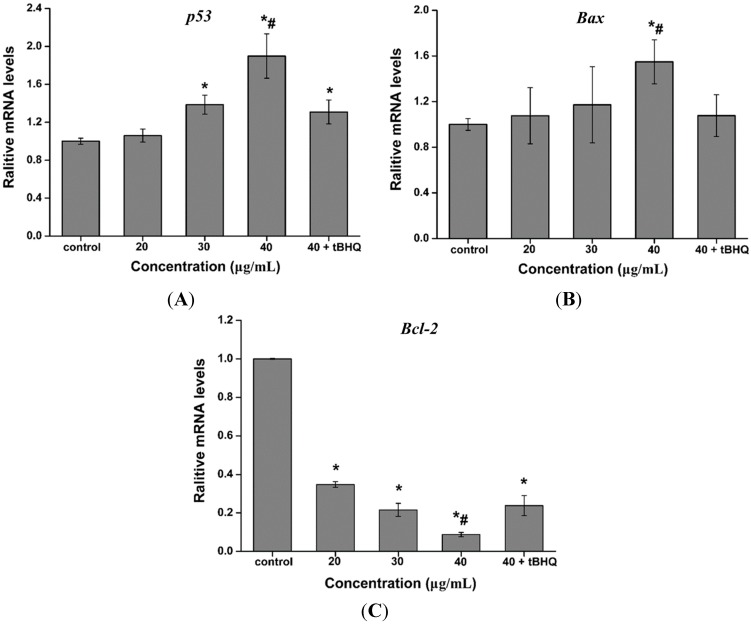
The mRNA expression levels of (**A**) *p53*, (**B**) *Bax* and (**C**) *Bcl-2* in AML 12 cells exposed to 20, 30, 40 μg/mL CdTe QDs and (40 μg/mL CdTe QDs + tBHQ) for 24 h. Data are presented as the mean ± SD from three independent experiments. The asterisks (*) and indicate statistically significant differences compared to control with *p* < 0.05, respectively. The number (#) signs indicate statistically significant differences compared to the tBHQ pre-treated group (*p* < 0.05).

Quantitative real-time PCR was used to analyze the mRNA levels of apoptotic genes (*p53*, *Bax* and *Bcl-2*) in AML 12 cells exposed to 0, 20, 30 and 40 µg/mL of CdTe QDs for 24 h, as well as, AML 12 cells exposed to 40 μg/mL CdTe QDs + tBHQ for 24 h, to determine if CdTe QDs induced apoptosis. The results showed that CdTe QDs significantly altered the expression levels of mRNA of these genes in AML 12 cells. The mRNA expression level of apoptotic genes *Bax* and *p53* were significantly up-regulated, while the expression of anti-apoptotic gene *Bcl-2* was significantly down-regulated in CdTe QDs treated cells compared to controls (Figure 6) (*p* < 0.05 for each). Pretreatment of cells with the antioxidant tBHQ for 24 h followed by CdTe QDs treatment for 24 h resulted in a significant attenuation of *Bax* and *p53* gene expression, and up-regulated of *Bcl-2* gene expression compared to CdTe QDs alone (Figure 6), confirming that CdTe QDs-induced apoptotic gene expression is mediated by oxidative stress.

### 2.8. Effect of CdTe QDs on the Expression of Nrf2 Signal Pathway Proteins

**Figure 7:**
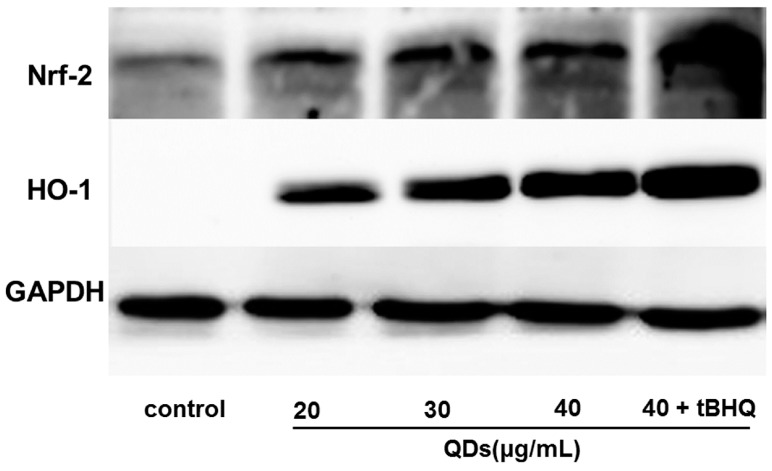
The protein expression level of Nrf2 and HO-1 in the total cellular protein extract or the nuclear extract of AML12 cells. AML12 cells were exposed to 20, 30, 40 µg/mL CdTe QDs and (40 µg/mL CdTe QDs + tBHQ) for 24 h. The protein expression of HO-1 was measured in the total cell lysates; whereas Nrf2 was measured in the nuclear fraction.

To evaluate if the elevated apoptotic genes transcript levels resulted in increased Nrf2 signal pathway proteins levels, the Nrf2 and HO-1 protein levels in AML 12 cells after exposure to CdTe QDs was investigated by western blot analysis, using GAPDH as an internal reference and loading control. A clear up-regulation of both Nrf2 and HO-1 protein levels are observed in the CdTe QDs-treated AML 12 cells, Nrf2 was significantly up-regulated 24 h after the initial exposure, while HO-1 was up-regulated after 24 h of CdTe QDs exposure (Figure 7). In addition, the translocation and accumulation of the Nrf2 protein in the nucleus of CdTe QDs-mediated stressed cells, required for its transcription factor functions, was observed by increased Nrf2 levels in the nuclear extract, but not in the total protein extract, representing the nuclear translocation of the Nrf2 protein after CdTe QD treatment. In AML 12 cells exposed to 20, 30, or 40 μg/mL, as well as 40 μg/mL + tBHQ, CdTe quantum dots for 24 h, the total Nrf2 and HO-1 protein increased as CdTe QD exposure concentration increased compared with the control group. In addition, the tBHQ intervention group had an increase in total cellular HO-1 expression compared to the nonintervention group.

## 3. Discussion

In a recent study using well-characterized CdTe QDs, we attempted to reduce CdTe QDs toxicity in L929 cells by controlling the exposure dose [24]. Several subsequent *in vivo* pharmacokinetic studies revealed that QDs are more likely to accumulate in the liver, spleen, and kidneys after intravenous injection, though there were few studies regarding the potential mechanisms of hepatocyte toxicity for the QDs. Here, we extended our previous work using mice and AML 12 cells to model potential mechanisms of hepatocyte toxicity after exposure to CdTe QDs.

The first indication of a toxic effect from intravenously administered CdTe QDs was a significant increase in the liver/bw ratio with a dose of 16.5 mg/kg.bw of CdTe QDs (Table 3). Organ weight ratio provides visual evidence of toxicity but is not quantitative. To quantify the liver toxicity of CdTe QDs, the next important step is an assessment of the standard biochemical markers of the liver organ. By measuring a variety of factors in the serum, it is possible to assess liver function, hepatocellular injury, and cholestasis. In the present study, the specificity of biochemical parameters associated with cellular damage in the liver, such as ALT, was not changed. Factors such as LDH and ALB were slightly changed, and a decrease of serum LDH activity in all exposed groups could be attributed to the increased energy consumption through transaminase reactions, glycolytic and oxidative pathways of glucose and alkaline phosphatase activity, rather than LDH activity. Additionally, the significant rise in ALB in CdTe QDs exposed groups indicates that synthesized proteins were liberated as a result of cytolysis, which is associated with the progression of the hepatoxic effect of these metals [25]. It has been suggested that it may be a result of oxidative stress, which occurs in the CdTe QDs toxicity. In other words, the reduced antioxidant production was due to the increased oxygen metabolites and the elevated free radicals, which caused a decrease in the activity of the antioxidant defense system [26].

Oxidative stress is currently believed to be the main modulator of adverse effects upon exposure to nanoparticles [13,27,28,29]. Previous studies have shown that exposure to nanoparticles could induce intracellular oxidative stress by disturbing the balance between the oxidant and antioxidant processes [30]. However, not much is known regarding the oxidative stress caused by QDs *in vivo*. Thus, to shed light on the mechanism responsible for hepatic damage induced by CdTe QDs, we evaluated the biomarker for lipid peroxidation (levels of MDA) and antioxidant processes (the activities of SOD and CAT) stimulated by QDs in the livers of mice. SOD is generally regarded as the primary line of defense against tissue and cellular damage caused by ROS. It catalyzes the dismutation of superoxide anion to peroxide. The liver also possesses an array of cellular defense systems, including antioxidant enzymes such as SOD and CAT [31,32]. CAT provides a second line of defense by dismutating peroxide into water and molecular oxygen [33]. In our study, exposure to CdTe QDs caused a significant increase in MDA at high dose treatments and resulted in a significant increase in SOD and CAT activities in liver tissues after treatment with 4.125 mg/kg.bw. Our findings suggest that intravenous exposure to CdTe QDs can deplete antioxidants in the liver, which may further cause an imbalance between oxidants and antioxidants, resulting in oxidative stress in the liver. This result indicated that CdTe QDs exposure via intravenous administration significantly increased the level of lipid peroxides marker malondialdehyde (MDA) in liver and activated the liver antioxidant system.

To further study the hepatotoxicity and regulatory mechanisms caused by CdTe QDs exposure at the cellular level, we explored the cytotoxicity of CdTe QD exposure in AML 12 cells. Initial work showed that CdTe QD effects occurred in a concentration- and time-dependent manner, consistent with our previous findings using the same source of CdTe QDs [24]. Considering that viability assays are vital steps in toxicology that explain the cellular response to a toxicant and that they give information on cell death, survival, and metabolic activities, we have exploited the high sensitivity of luminescence- and fluorescence-based assays to study the cytotoxicity of CdTe QDs. The ATP concentration is described as the product of the viable cell number and the average amount of ATP produced by each viable cell. The result showed that the ATP concentration in AML 12 cells greatly decreased as the concentration of CdTe QDs increased. These results indicated that the bioenergy crisis (ATP depletion) should be involved with the massive death of AML 12 cells observed after exposure. While the CdTe QDs used here are not identical to those used in other studies, these results are consistent with past work using different cell lines [7,14,15]. Chan *et al.* [7] showed that treatment of IMR-32 cells with CdSe QDs caused changes in bioreduction of MTT in a concentration-dependent manner, and they proposed that the increase in ROS and mitochondrial-dependent apoptotic processes were responsible for the cytotoxicity of the CdSe QDs. Additionally, Nguyen *et al.* [14,15] showed that CdTe QDs caused cytotoxicity in HepG2 cells in a time- and dose-dependent manner after treatment with 0.001–1 μg/mL for 6–24 h, and the authors demonstrated that CdTe QDs induced multifarious toxicity by producing ROS, changing the mitochondrial structure, and inducing apoptosis. These previous studies suggested that toxicity of CdTe QDs could be due to the ROS formation, but more details about possible mechanisms needed to be clarified. It is worth noting that CdTe QD effects occurred in a concentration- and time-dependent manners *in vitro*, but *in vivo* results did not change dose-dependently (e.g., LDH, SOD, CAT). Sometimes, however, compounds that show very high activity *in vitro* may prove later to have no *in vivo* activity, or to be highly toxic in *in vivo* models. Lack of *in vivo* activity may be attributed to different biological environments, and the differences of toxic trends may result from the formation of reactive metabolites. Meanwhile, the liver is an important biosynthetic organ where ROS are produced in substantial amounts as side products of energy production in the mitochondrial electron transport chain or as the result of biosyntheses involving diverse oxygenases [34]. When isolated liver cells are exposed to CdTe QDs, toxic effects are observed only if a concentration of CdTe QDs is used that is at least ten times higher than the level that causes liver damage *in vivo*. So inconsistency of the dose-response data might be observed *in vivo* and *in vitro*.

To investigate the factors that are possibly responsible for CdTe-induced cytotoxicity in greater detail, intracellular ROS production was measured through the production of DCF using the fluorescent dye dihydroethidium (DHE), which is a specific probe to indicate presence of O^2^^−^. In our previous study, we found that QDs induced cytotoxicity in L929 cells through changes in ROS generation and mitochondrial membrane potential [24]. The present study showed that CdTe QDs treated cells exhibited an increase in ROS formation, which confirms findings from previous studies that showed ROS generation from CdTe QD exposures [16,35]. In the tBHQ pre-treatment group, the ROS levels increased less in the AML 12 cells after exposure to 40 μg/mL QDs for 24 h. These results clearly showed that the protective effect of tBHQ against oxidative stress was due to the decrease in ROS induced by CdTe QDs. ROS was generated in the presence of CdTe QDs, which could explain the endogenous antioxidant defense system disturbances and other toxicological outcomes. In addition, ROS has been postulated to be closely related to cell apoptosis that is activated by nanoparticles [36,37]. High levels of ROS can cause damage to macromolecules and further induce cell apoptosis [38].

To further study the cytotoxicity initiated by CdTe QDs, we investigated apoptosis using CdTe QDs in AML 12 cells. Annexin V-FITC/PI staining showed distinct dose-dependent induction of apoptosis after 24 h in AML 12 cells treated with CdTe QDs. Furthermore, there are two major apoptotic pathways, *i.e.*, the extrinsic pathways (death receptors) and intrinsic pathways (mitochondria), which are initiated by the caspase family of proteins. Thus, we examined the expression of p53, the most thoroughly studied factor involved in the mitochondrial signaling pathway. This approach was validated by the ability of CdTe QDs to increase p53 expression. Interestingly, expression of p53 was increased after CdTe QDs treatment. Links between ROS and p53 activity have been previously reported. The results demonstrated that activated p53 increases cellular ROS by enhancing the transcription of pro-apoptotic genes. At the same time, the anti-apoptotic gene Bcl-2 was up-regulated in AML 12 cells as a result of CdTe QD exposure. Taken together, the up-regulation of p53 leads to the activation of pro-apoptotic member Bax, which induces permeabilization of the outer mitochondrial membrane, resulting in the release of soluble proteins from the intermembrane space into the cytosol, where they promote caspase activation [39,40,41]. Interestingly, tBHQ pre-exposure resulted in significant decrease in the percentage of apoptosis, the down-regulated gene expression of p53 and Bax, and the up-regulated expression of Bcl-2. These results suggest that QDs can potentially change apoptotic protein expression and trigger apoptosis in mitochondria-dependent pathways in AML 12 cells [42]. The efficient protection of tBHQ against apoptosis in AML 12 cell led us to hypothesize that tBHQ’s beneficial action in antioxidant defense system contributed to the reduction of the cytotoxicity of CdTe QDs to AML 12 cells.

As underlying mechanisms of cytotoxicity, our results suggest that CdTe QDs may exert their toxicity through ROS generation apoptosis induced in a mitochondria-dependent manner. These reports raise a possibility that the antioxidant defense system may be required to counteract oxidative stress and consequent cytotoxicity by CdTe QDs. Because the transcription factor Nrf2 can therefore be considered as a master switch for antioxidant defense, serving as a functional indicator of oxidative insult caused by nanomaterials [43], one hypothesis could be that Nrf2 may affect the sensitivity of cells to CdTe QDs toxicity. Based on our results, we have explored the potential role of Nrf2 in CdTe QDs toxicity in AML 12 cells. HO-1, one of the antioxidant proteins regulated by Nrf2, has long been identified as proto-oncogenic due to its anti-apoptotic and proangiogenic properties [44]. With this in mind, we explored the potential role of Nrf2 and HO-1 in oxidative damage and apoptosis induction by CdTe QDs. AML 12 cells and tBHQ pre-treated AML 12 cells were used in the present study. tBHQ was widely used as a Nrf2 activator and confers protection against cellular dysfunction induced by oxidative stress inducers via an Nrf2-dependent mechanism [45,46]. In tBHQ pre-treatment group, the ROS generation and apoptosis levels increased less than in nonspecific control cells after same CdTe QD treatment. Furthermore, pre-treatment with tBHQ in AML 12 cells resulted in a significant increase in Nrf2 and HO-1 levels compared to nonspecific control cells. These suggest that the ROS-counteracting Nrf2 system is important for the protection of cells from QD toxicity.

## 4. Experimental Section

### 4.1. Preparation and Characterization of Quantum Dots

The quantum dots (QDs) used in this study were 2.2 nm in size and synthesized by the Department of Biomedical Engineering, Southeast University, Nanjing, China. Briefly, CdTe precursors were generated using an applied potential of 1.2 V in a electrolyte containing 2.0 mmol/L CdCl_2_ and 16.6 µL 3-mercaptopropionic acid (MPA) at pH 10 adjusted with 0.1 mol/L NaOH. The solution of CdTe precursors was then heated in a water bath with moderate stirring at 80 °C. The CdTe QDs were gradually crystallized, and their size was controlled by changing the heating time to 2 h to obtain 2.2 nm sized CdTe QDs. The resulting CdTe QDs were deposited by acetone, and after centrifugation, the precipitate was washed with acetone at least three times and re-dissolved in water. More details are available in a previously published report [47].

The transmission electron microscopy (TEM) image was taken by a JEM 2100 microscope (JEOL, Tokyo, Japan) with an acceleration voltage of 200 kV. The absorption and emission spectra were recorded with a UV3150 scanning spectrophotometer (Shimadzu, Kyoto, Japan) and an F-7000 Spectrofluorometer (Hitachi, Tokyo, Japan), respectively. The particle size and zeta potential of the QDs in pure water and DMEM/F12 medium were measured with a Malvern Zetasizer (Nano-ZS, Malvern Instruments, Worcestershire, UK).

### 4.2. In Vivo Sample Preparation

Four-week-old male Institute of Cancer Research (ICR) mice (18–22 g) were provided by the Yangzhou University Comparative Medicine Center, Yangzhou, China. Animals were bred under specific pathogen free (SPF) conditions and barrier maintained during the experiment. They were maintained in animal care facilities fully accredited by the China Food and Drug Administration in a temperature-regulated room (temperature of 22 ± 1 °C, humidity of 60% ± 10% and light/dark cycle of 12 h/12 h) for five days prior to the study. Nutritionally complete laboratory food and water were provided ad libitum. All animal experiments were performed in compliance with the local ethics committee (Science&Technology Department of Jiangsu Provincial Government, SYXK(SU) 2010-0004, 18 June 2010).

ICR mice were randomly divided into four groups according to weight with each group comprising of four mice. ICR mice were randomly divided into four groups: a control group and three QDs treatment groups (4.125, 8.25 and 16.5 mg/kg body weight, respectively). Suspensions of QDs were administered to the mice via intravenous administration at a dose of 0.1 mL/10 g body weight, once a week for four weeks. Control mice were treated with normal saline (NS). On the 28th day, the animals were sacrificed, after being anesthetized with ether, to enable further assessment of the biochemical parameters.

### 4.3. Clinical Observations and Pathological Examinations

The animals were observed daily for clinical signs and mortality. Body weight was measured weekly throughout the study period. At the end of the four-week exposure period, all of the mice were necropsied. Blood was collected for blood biochemistry from the abdominal aorta of mice under pentobarbital anesthesia after overnight fasting. After being collected and stored at 20 °C, the serum samples were characterized on a Biochemical Autoanalyzer (Type 7170, Hitachi, Tokyo, Japan) for the following clinical chemical parameters: total protein (TP), total bilirubin (TBIL), direct bilirubin (DBIL), alanine transaminase (ALT), aspartate aminotransferase (AST), γ-glutamyl transpeptidase (γ-GT), alkaline phosphatase (ALP), albumin (ALB) and lactate dehydrogenase (LDH). For histological observations, the formalin-fixed livers were embedded in paraffin, thin-sectioned, and then mounted on glass microscope slides using standard histopathological techniques. The mounted sections were stained with hematoxylin-eosin (H&E) and examined by light microscopy.

### 4.4. Oxidative Stress Markers and Enzyme Activity Assays Analysis

For the assays of malondialdehyde (MDA) content, superoxide dismutase (SOD) and catalase (CAT) activity, the liver was minced and homogenized in 4 °C water three times (10 s/time, intermittently for 30 s) to yield 10% (*w*/*v*) homogenate. The homogenates were centrifuged at 2000 rpm for 10 min to obtain the supernatants. Protein concentrations in the supernatants were determined according to the Bradford method, using bovine serum albumin as the standard [48]. The MDA level, SOD activity, and CAT activity in the livers were assessed according to the manufacturer’s instructions (Nanjing Jiancheng Biotechnology Institute, Nanjing, China). Briefly, the level of MDA was expressed as nmol MDA/mg protein, using 1,1,3,3-tetraethoxypropane (TEP). SOD activity was determined by the decrease in absorbance at 340 nm due to NADPH oxidation (by the chemically generated superoxide anion) compared to reaction blanks with no SOD activity. One unit of SOD activity was defined as the amount of enzyme that inhibited the oxidation of NADPH by 50%. CAT activity was measured by monitoring the decrease of absorbance at 240 nm due to H_2_O_2_ decomposition. One unit of CAT activity was defined as the amount of enzyme that catalyzed the conversion of 1 μmol of H_2_O_2_ in a minute.

### 4.5. Cell Culture and Treatments

AML 12 cells (murine hepatocyte cell line) were purchased from Shanghai Institute of Cell Biology, Chinese Academy Sciences, Shanghai, China. AML 12 cells were some of the first and most widely used cells in cytotoxicity tests. AML 12 cells used in the cytotoxicity tests were maintained in DMEM/F-12 medium supplemented with 10% FBS, penicillin 100 IU/mL, and streptomycin 100 μg/mL (Gibco, Carlsbad, CA, USA), and they were cultured at 37 °C in a 5% CO_2_ humidified incubator. Cells in the logarithmic growth phase were used in all the experiments. The test suspension of CdTe QDs were prepared using the culture media and dispersed for 10 min using a sonicator (Branson Inc., Danbury, CT, USA) to prevent aggregation. The cells were treated with various concentrations of particles according to the time schedule designated in the following section for each toxicological study. All measurements were conducted in duplicate in three independent experiments.

### 4.6. Viability Analysis

Cell viability was measured using the 3-[4,5-dimethylthiazol-2-yl]-2,5-diphenyltetrazolium bromide (MTT) colorimetric assay. AML 12 cells were grown until they reached 80% confluency. The cells were then harvested and seeded into 96-well culture plates at a density of 8000 cells/well in a total volume of 200 µL and allowed to attach and grow for 24 h. The supernatant in each well was then replaced with DMEM/F-12 medium containing various concentrations of CdTe QDs: 0, 27.66, 41.49, 53.94, 70.12, 91.16 and 118.50 μg/mL. After 24 and 48 h of incubation, 100 µL of MTT (Sigma-Aldrich, Shanghai, China) was added to each well. After 4 h of incubation, the supernatant was removed and 150 µL dimethylsulfoxide (DMSO, Sigma-Aldrich, Shanghai, China) was added to each well. Samples were then shaken for 15 min at 37 °C to solubilize the formazan products. Absorbance was quantified at a wavelength of 490 nm using a POLARstar OPTIMA microplate reader (Mithras LB940, Berthold, Germany). Cell viability was expressed as the ratio between the amounts of formazan measured in treated cells to amounts measured in cells of non-treated control group. All experiments were performed in triplicate.

### 4.7. Determination of Cellular ATP Levels

The ATP level was measured by the luciferin-luciferase method following the protocol of the ATP assay kit (CellTiter-Glo luminescent cell viability Assay, Promega, Madison, WI, USA). After 24 and 48 h of incubation with CdTe QDs at different concentrations, cells were washed twice and then resolved with ATP lysis buffer on ice. The ATP concentration in the cell lysate was measured using a POLARstar OPTIMA microplate reader (Mithras LB940, Berthold, Stuttgart, Germany).

### 4.8. Determination of Intracellular Reactive Oxygen Species (ROS) Generation

The changes in the levels of ROS developed inside the cells in presence of QDs were analyzed by following the method of Zhang *et al.* using 2ʹ,7ʹ-dichlorfluorescein-diacetate (DCFH-DA) [49]. AML 12 cells were plated in a six-well culture plate at a density of 1.5 × 10^5^ cells/mL with 2 mL/well for 2 h. Then, the medium was discarded, and the cells were washed with phosphate buffered saline (PBS). The tBHQ pretreatment group was treated with 10 μmol/L tBHQ. The remaining group had medium containing 10% FBS added and were cultured for 12 h. Then, the cells were treated with 20, 30 and 40 µg/mL of QDs for 24 h. Thereafter, the cells were harvested and washed with PBS and then loaded with 10 µM DCFH-DA diluted in serum-free medium and incubated at 37 °C for 30 min. All samples were rinsed three times with serum-free medium, and then resuspended in PBS. The fluorescence was measured using flow cytometry at the excitation and emission wavelengths of 488 and 525 nm for dichlorofluorescein (DCF) fluorescence, respectively. The mean fluorescence intensity (MFI) of the DCF channel was determined using CellQuest Pro software (BD Biosciences, San Jose, CA, USA). The results were expressed as the fold change of the percentage increase in DCF channel

### 4.9. Annexin V-FITC/Propidium Iodide Apoptosis Assay

Normal, apoptotic, and necrotic cells were distinguished using an Annexin V-FITC/propidium iodide assay kit (KeyGEN Biotech, Nanjing, China) according to the manufacturer’s instructions. AML 12 cells were plated in a six-well culture plate at a density of 1.5 × 10^5^ cells/mL with 2 mL/well for 12 h. Then, the medium was discarded, and the cells were washed with PBS. The tBHQ pretreatment group was treated with 10 μmol/L tBHQ. The remaining group had medium containing 10% FBS added and were cultured for 12 h. Then, the cells were treated with 20, 30 and 40 µg/mL of QDs for 24 h. Thereafter, the cells were harvested, washed with PBS, and resuspended in 400 µL of binding buffer to a density of 1 × 10^6^ cells/mL, and 5 µL of Annexin V-FITC was then added to the samples. After incubation for 15 min at 4 °C in the dark, 10 µL of propidium iodide was added to the samples, and the cells were incubated for 5 min. Cells were analyzed by flow cytometry using a 4-color FACSCalibur (BD Biosciences, San Jose, CA, USA) for fluorescence within 15 min.

### 4.10. Real-Time Quantitative Analysis

AML 12 cells were plated in a six-well culture plate at a density of 5 × 10^5^ cells/mL with 2 mL/well for 24 h. Then, the cells were treated in accordance with the method of the assay [49]. The total RNA was extracted using the Trizol protocol (Invitrogen, Carlsbad, CA, USA). cDNA was synthesized from the total RNA using Super Script III Reverse Transcriptase (Invitrogen). Real-time quantitative RT-PCR analysis was performed with an automated sequence detection system (ABI Prism 7300, Tokyo, Japan). Expression of mouse genes *GAPDH*, *Bax*, *Bcl-2*, and *p53* were analyzed using SYBR Green Premix Exaq II (TOYOBO, Osaka, Japan). Primers were designed using Primer Express Software (Applied Biosystems, Foster, CA, USA) according to guidelines (Table 4). The real-time PCR data were analyzed using the 2^−ΔΔ*C*t^ relative quantitation method, using the manufacturer’s standards of GAPDH [50,51].

**Table 4:** Primer sequences of apoptosis-related genes used in this study.

Gene Primer	Sequence (5ʹ-3ʹ)	Product Size (bp)
*Bax*	Forward: AGACAGGGGCCTTTTTGCTAC	137
	Reverse: AATTCGCCGGAGACACTCG	
*Bcl-2*	Forward: GAGAGCGTCAACAGGGAGATG	108
	Reverse: CCAGCCTCCGTTATCCTGGA	
*p53*	Forward: CCCCTGTCATCTTTTGTCCCT	137
	Reverse: AGCTGGCAGAATAGCTTATTGAG	
*GAPDH*	Forward: AGGTCGGTGTGAACGGATTTG	123
	Reverse: TGTAGACCATGTAGTTGAGGTCA	

### 4.11. Western Blot Analysis of Nrf2 and HO-1 Activation

AML 12 cells were plated in a six-well culture plate at a density of 5 × 10^5^ cells/mL with 2 mL/well for 24 h. Then, the cells were treated in accordance with the 1.5 assay method. For total protein extraction, the cells were lysed in a buffer containing complete protease inhibitor mixture. After centrifugation, 20 µg of total proteins were electrophoresed in 10% polyacrylamide gels and transferred to an ECL membrane. Immunoblotting was carried out with an anti-Nrf2 polyclonal antibodies purchased from Cell Signaling (Beverly, MA, USA) and an anti-HO-1 monoclonal antibody purchased from Abcam (Victoria, BC, Canada) in phosphate-buffered saline (PBS) with 0.2% Tween 20 and 5% BSA. After washing, the membrane was probed with horseradish peroxidase-conjugated goat antiserum to rabbit (Chemicon, Beverly, MA, USA) and developed by the enhanced chemiluminescence method (Amersham Biosciences, Piscataway, NJ, USA). Three replicates for each treatment and a control were conducted for the western blot analysis.

### 4.12. Statistical Analysis

All data were expressed as the mean ± standard deviation. The results were compared by one-way analysis of variance (ANOVA) followed by Dunnett’s test for comparison of treatment groups to the negative control group and Least Significant Difference (LSD) test for pairwise comparisons among treatment groups. A value of *p* < 0.05 was considered as statistically significant. 

## 5. Conclusions

Here, the oxidative stress in the livers of mice and AML 12 cells exposed to CdTe QDs was measured, and the potential mechanisms of toxicity were evaluated. CdTe QD exposure via intravenous administration significantly increased the level of lipid peroxides marker, MDA, in the liver. After treatment with CdTe QDs, AML 12 cells showed an increase in ROS generation and apoptosis, with a concomitant increase in the gene expression of tumor suppressor gene *p53* and pro-apoptotic gene *Bcl-2*, as well as a decrease in the anti-apoptosis gene expression *Bax*, suggesting that a mitochondria-mediated pathway is involved in CdTe QD-induced apoptosis. Finally, we showed that Nrf2 deficiency blocked induced oxidative stress to protect cells from injury induced by CdTe QDs. Taken together, our results demonstrate that tBHQ-mediated antioxidant response can prevent CdTe QDs-induced oxidative stress and AML 12 cell apoptosis. These findings provide insights into the regulatory mechanisms involved in the activation of Nrf2 signaling that confers protection against CdTe QDs-induced apoptosis in hepatocytes.
